# INTEREST GROUP SESSION—GERIATRIC EDUCATION: AGHE PROGRAM OF MERIT: PRACTICAL AND TECHNICAL APPLICATION INFORMATION AND ASSISTANCE

**DOI:** 10.1093/geroni/igz038.2113

**Published:** 2019-11-08

**Authors:** Marilyn R Gugliucci, Shannon D Mathews, Pamela Elfenbein

**Affiliations:** 1 University of New England College of Osteopathic Medicine, Biddeford, Maine, United States; 2 Winston Salem State University, Winston Salem, North Carolina, United States; 3 University of North Georgia, Oakwood, Georgia, United States

## Abstract

At a time in education when it is important for programs at colleges and universities to be productive in teaching, student recruitment, and income generation, the AGHE Program of Merit review process can aid in advancing gerontology/geriatrics programs. The Program of Merit has expanded its review now Colleges of Osteopathic Medicine and any Health Professions program may apply for Program of Merit status – your institution need not be an AGHE member to apply. The POM designation provides gerontology programs and those health disciplines that include geriatrics/gerontology content in the curriculum with an AGHE “stamp of approval” which can be used to verify program quality to administrators, to lobby for additional resources, to maintain a quality program, to market the program, and to recruit prospective students into the program. This session will begin with the “Why,” “What” and “How” of applying for Program of Merit and then provide time for small group consultation regarding the Program of Merit application. Preparation of the self-study document will be detailed by the experienced faculty members followed by the review process and timelines associated with application process. Free consultation available!

